# Comparative genome features and secondary metabolite biosynthetic potential of *Kutzneria chonburiensis* and other species of the genus *Kutzneria*

**DOI:** 10.1038/s41598-023-36039-x

**Published:** 2023-05-31

**Authors:** Manee Chanama, Pinidphon Prombutara, Suchart Chanama

**Affiliations:** 1 Department of Microbiology, Faculty of Public Health, Mahidol University, Bangkok, 10400 Thailand; 2 Omics Sciences and Bioinformatics Center, Faculty of Science, Chulalongkorn University, Bangkok, 10330 Thailand; 3 Department of Biochemistry, Faculty of Science, Chulalongkorn University, Bangkok, 10330 Thailand

**Keywords:** Bacteria, Comparative genomics, Phylogenomics

## Abstract

Actinobacteria are well known as a rich source of diversity of bioactive secondary metabolites. *Kutzneria*, a rare actinobacteria belonging to the family *Pseudonocardiaceae* has abundance of secondary metabolite biosynthetic gene clusters (BGCs) and is one of important source of natural products and worthy of priority investigation. Currently, *Kutzneria chonburiensis* SMC256^T^ has been the latest type-strain of the genus and its genome sequence has not been reported yet. Therefore, we present the first report of new complete genome sequence of SMC256^T^ (genome size of 10.4 Mbp) with genome annotation and feature comparison between SMC256^T^ and other publicly available *Kutzneria* species. The results from comparative and functional genomic analyses regarding the phylogenomic and the clusters of orthologous groups of proteins (COGs) analyses indicated that SMC256^T^ is most closely related to *Kutzneria* sp. 744, *Kutzneria kofuensis, Kutzneria* sp. CA-103260 and *Kutzneria buriramensis*. Furthermore, a total of 322 BGCs were also detected and showed diversity among the *Kutzneria* genomes. Out of which, 38 clusters showing the best hit to the most known BGCs were predicted in the SMC256^T^genome. We observed that six clusters responsible for biosynthesis of antimicrobials/antitumor metabolites were strain-specific in *Kutzneria chonburiensis*. These putative metabolites include virginiamycin S1, lysolipin I, esmeraldin, rakicidin, aclacinomycin and streptoseomycin. Based on these findings, the genome of *Kutzneria chonburiensis* contains distinct and unidentified BGCs different from other members of the genus, and the use of integrative genomic-based approach would be a useful alternative effort to target, isolate and identify putative and undiscovered secondary metabolites suspected to have new and/or specific bioactivity in the *Kutzneria*.

## Introduction

Recent advances in high-throughput whole genome sequencing, genome mining and secondary metabolite biosynthetic gene clusters (BGCs) analyses have led to discovery of numerous bioactive metabolites, e.g. new and diverse antimicrobial agents, from microbial sources^1–3^. The most diverse and multiple secondary metabolite gene clusters have been investigated in a group of actinomycetes genomes, particularly in more difficultly isolated and cultured subgroups namely rare actinomycetes^4–6^. One of them includes a species belonging to the genus *Kutzneria*. *Kutzneria* is a genus of bacteria in Phylum Actinobacteria, Order *Pseudonocardiales* and Family *Pseudonocardiaceae*^7^. They are non-motile, aerobic, mesophilic, thermotolerant, Gram-positive and chemoorganotrophs. The majority of this species have been isolated from soils in different habitats, including tropical rainforest, mountain forest, deciduous forest, rhizosphere and sediment. Members of this genus are usually associated with soil, but little is known about their role within this surroundings. They are probably involved in the primary decomposition of plant material in soils^7^. To date, there is information of 15 *Kutzneria* strains on public databases (five strains with systematic classification: *Kutzneria albida*, *Kutzneria viridogrisea*, *Kutzneria kofuensis*, *Kutzneria buriramensis* and *Kutzneria chonburiensis* and ten strains without taxonomic description: *Kutzneria* species strains CA-103260, 744, A14, TSII, TM-S116, TM-B149, RMD-3Y-3-1, RDB-177, 306G04 and L1988), of which seven strains (*Kutzneria albida*, *K. viridogrisea*, *K. kofuensis*, *K. buriramensis*, *K. chonburiensis*, *Kutzneria* species strains CA-103260 and 744) have their genome sequences and the remainder eight strains (*Kutzneria* species strains A14, TSII, TM-S116, TM-B149, RMD-3Y-3-1, RDB-177, 306G04 and L1988) have 16S rRNA gene sequences deposited in public databases (see Supplementary Table S1)^8^. Because there are hardly any complete genome assemblies of members of the genus *Kutzneria*, only 3 genomes obtained from *K. albida*, *K. chonburiensis* and strain CA-103260 were fully sequenced and the remaining incomplete genome sequences were of *K. buriramensis*, *K. viridogrisea*, *K. kofuensis* and strain 744^8^. With the recent improvement of DNA sequencing technology, the quality and completeness of bacterial genomes assemblies have increased dramatically^9^. As a result, development of a suite of bioinformatics tools which can be applied to annotate and mine these genomes has become increasingly essential. A number of bioinformatics tools have been developed, including BAGEL^10^, ClustScan^11^, CLUSEAN^12^, NP searcher^13^, PRISM^14^, and antiSMASH^15^, to identify BGCs within the genome. Most of these tools count on algorithms that map the highly conserved sequences within the BGCs to their location. Genome mining approaches enable prediction of BGCs from genome data rapidly and effortlessly, and accelerate the process linking the product with their corresponding BGCs.

First report in discovery of important secondary metabolites based on genome mining was investigated in *K. albida* DSM43870^T^. The antiSMASH, bioinformatics analysis tool for the rapid genome-wide identification, annotation and analysis of secondary metabolite BGCs, is able to identify BGCs responsible for the biosynthesis of aculeximycin that shows antimicrobial activity against Gram-positive bacteria, fungi and mosquito larvae^16^. Recently, the genome mining technique has also been applied to characterize BGCs in order to discover new 30-membered macrolides (epemicins A and B) in the culture of *Kutzneria* sp. CA-103260, which genome contains BGC similarly to the macrolide aculeximycin BGC found in *K. albida*^6^. These compounds showed antimicrobial activity against methicillin resistant *Staphylococcus aureus* (MRSA) and showed chemical structure related to aculeximycin core with some structural diversity. As a consequence, these findings present the potential of rare actinobacteria to produce new and structurally diverse metabolites. In this work, we therefore present the first report of complete genome sequence of *Kutzneria chonburiensis* SMC256^T^ by performing the genome sequencing using Oxford Nanopore MinION long read and Illumina short read sequencing technologies. Subsequently, the complete genome is subjected to annotation, functional and genomic features analyses. Furthermore, we carried out the comparative genome analysis of *K. chonburiensis* with other related *Kutzneria* species to obtain an in-depth comparison of metabolic potential of the representatives of this genus, and would allow to identify diversity and unique of the secondary metabolite biosynthetic gene clusters among them.

## Results

### Complete genome sequence characteristics of *Kutzneria chonburiensis* and its comparison to other rare *Kutzneria* species

**Figure 1:**
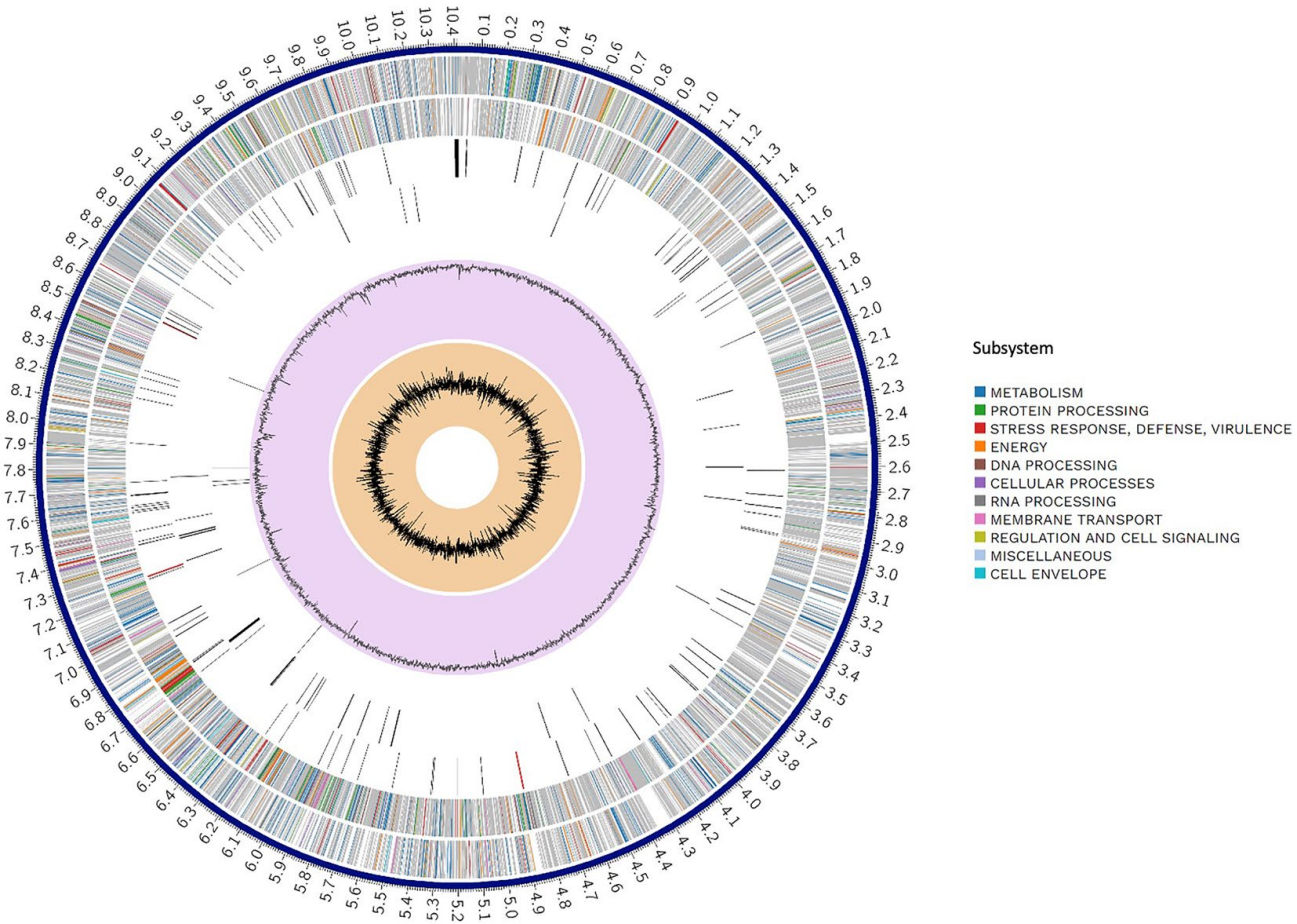
A circular map displays distribution of the genome annotations of *Kutzneria chonburiensis* strain SMC256^T^. From outer to inner rings: contig, CDS on the forward strand, CDS on the reverse strand, RNA genes, antimicrobial resistance genes, virulence factors, GC content and GC skew. The color codes of the CDS on the forward and reverse strand indicate the subsystem that these genes belong to.

**Figure 2:**
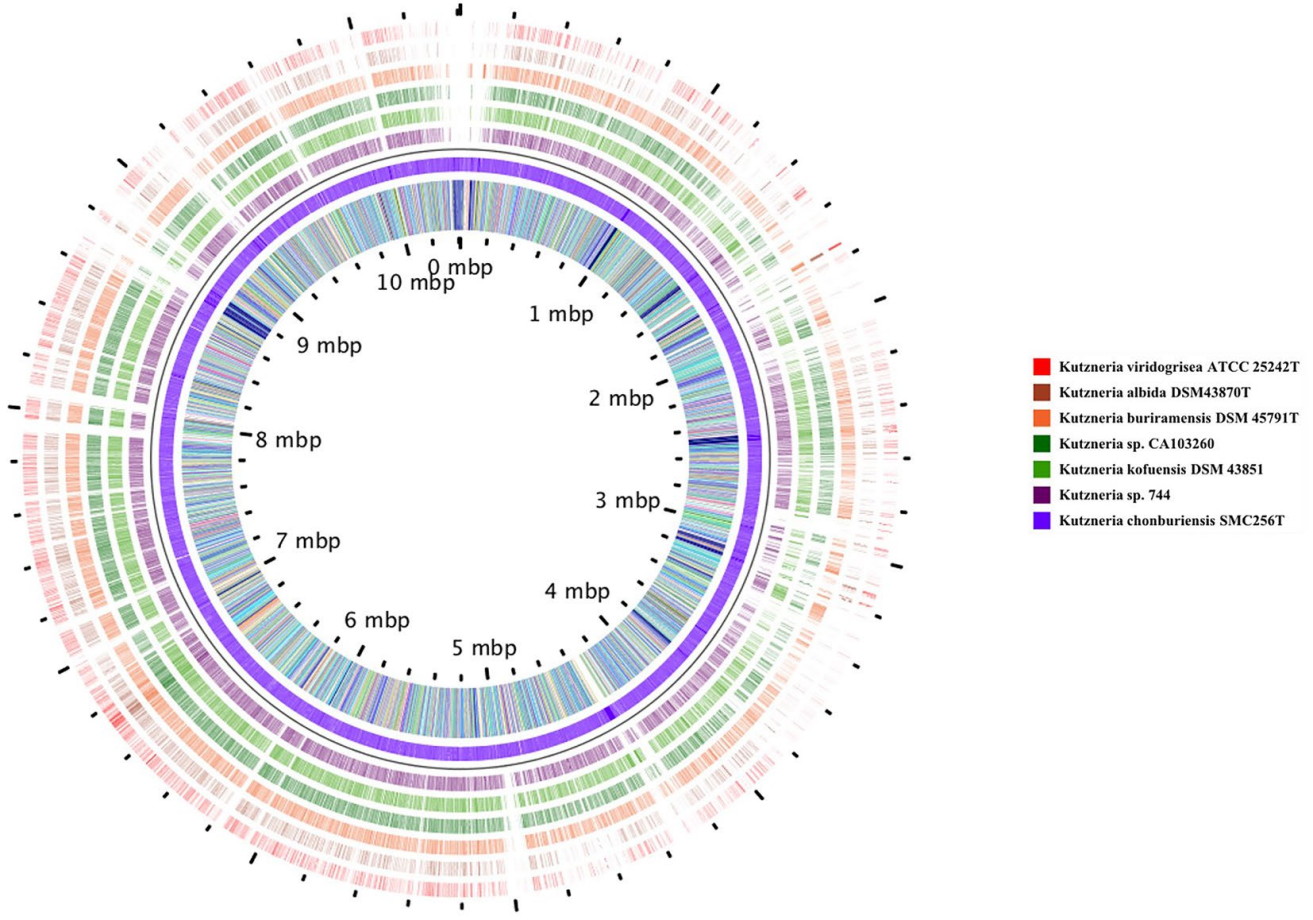
Genomic comparison of *Kutzneria chonburiensis* strain SMC256^T^ to other *Kutzneria* sp. genomes. The gaps and color shades in the circular map exhibit regions of no and low similarity, respectively. From outside: *Kutzneria viridogrisea*, *K. albida*, *K. buriramensis*, *Kutzneria* sp. CA103260, *K. kofuensis*, *Kutzneria* sp.744 and *K. chonburiensis*.

**Figure 3:**
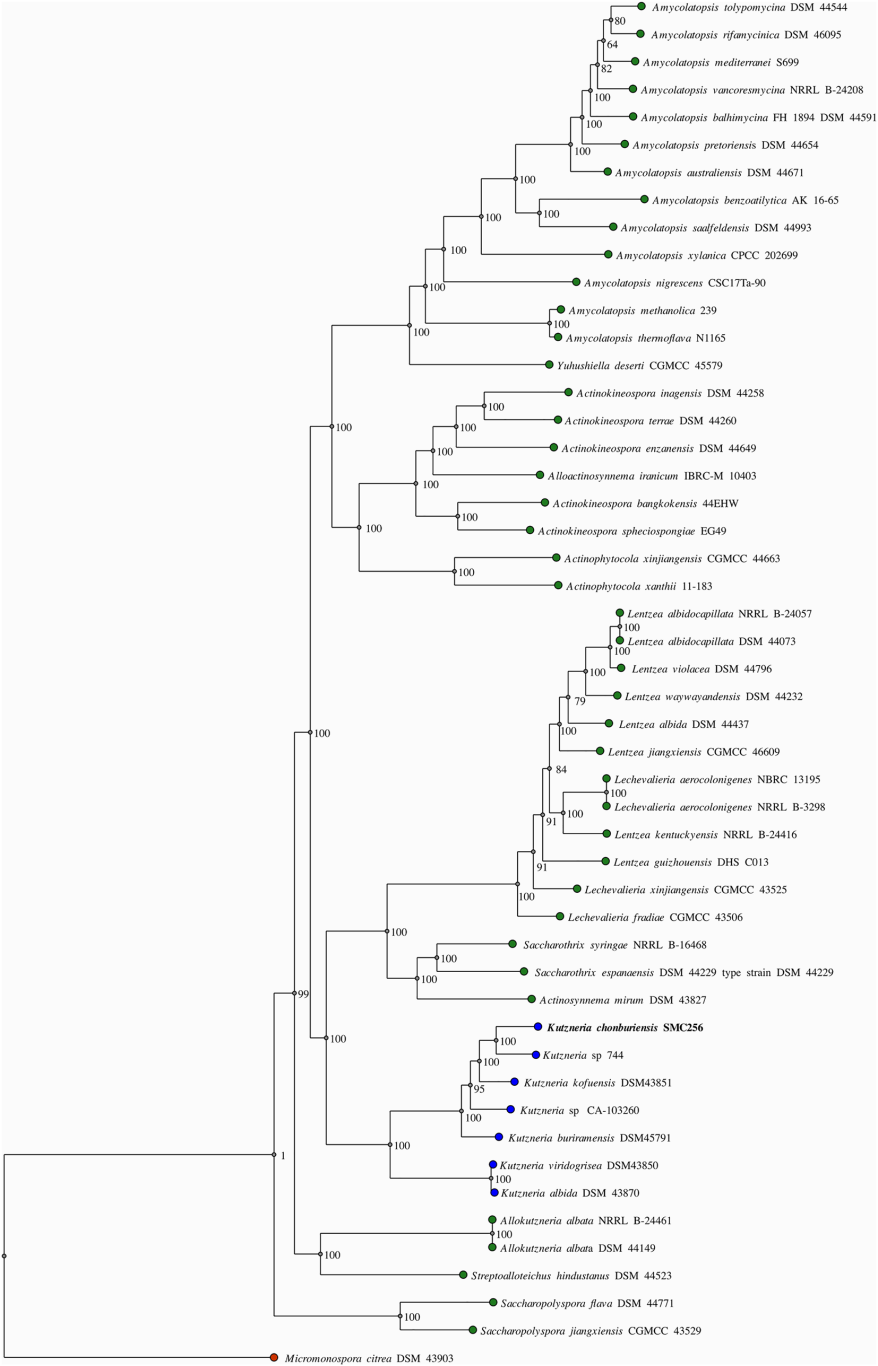
Phylogenomic tree for *Kutzneria* species and some members of the family *Pseudonocardiaceae*.

**Figure 4:**
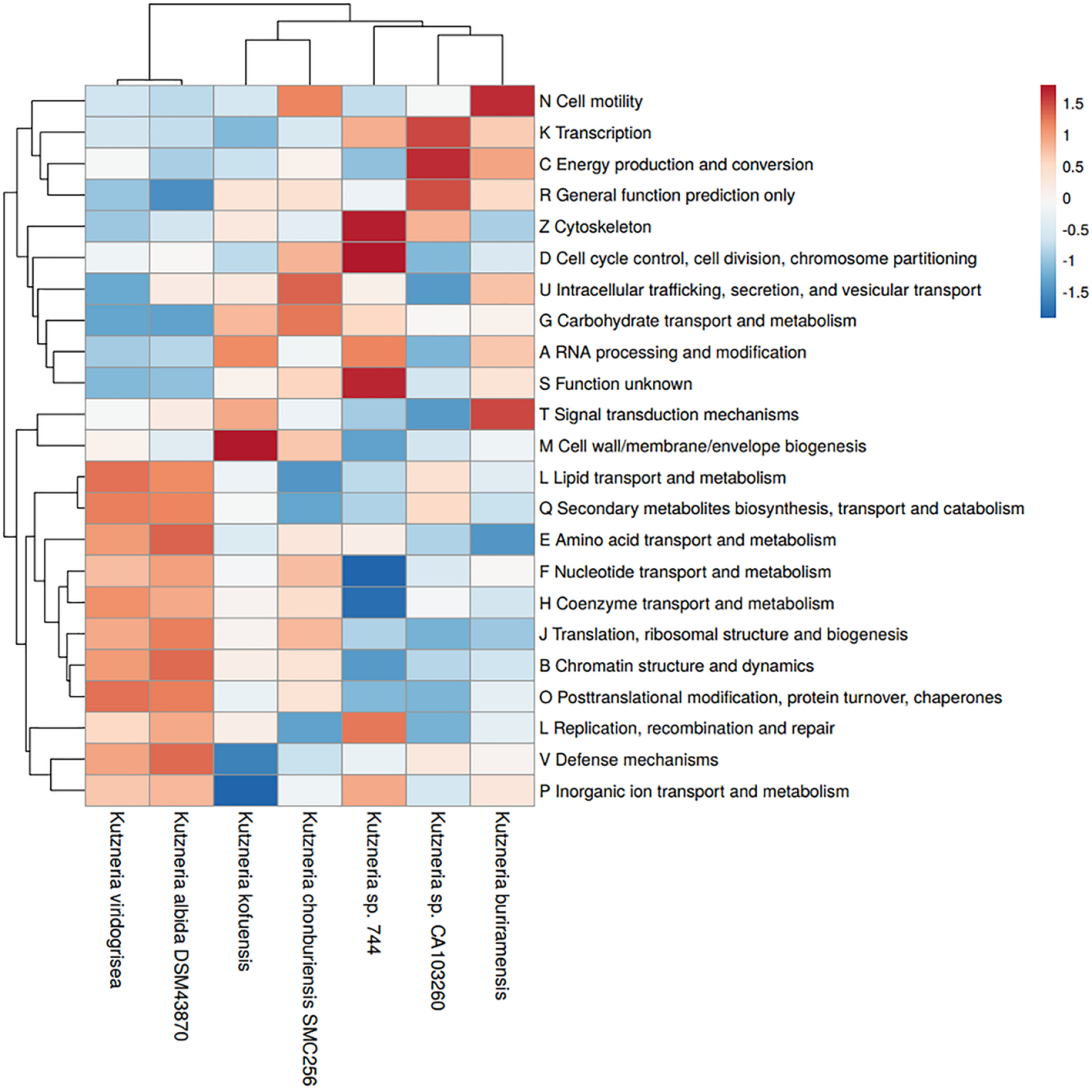
Functional classification of protein-coding genes presented in *Kutzneria* genomes based on the abundance of the clusters of orthologous groups of proteins (COGs). The color code represents the level of abundance.

The repeat regions obtained by short-read sequencing frequently make the step of assembly complicated and resulted in underestimation of the repetitive contents. To overcome this limitation of short reads, a hybrid strategy, utilizing sequence data generated by the long- and short-read sequencing methods, has been used in an attempt to complete genome assembly in the *Kutzneria chonburiensis* strain SMC256^T^. The complete sequenced genome of *K. chonburiensis* reveals a circular chromosome of 10,411,187 bp (10.4 Mbp) and GC content of 69.86% as depicted on a circular map (Fig. 1). The whole complete genome sequence is subject to be analyzed using the RAST server system to predict and assign functions to the genes and the results illustrated the total genes in the genome: 9,564 protein-coding sequences (CDS), 74 tRNA-coding genes, 9 rRNA coding genes, and 1 tmRNA genes (Table 1). At the present moment, only 7 sequenced genomes of *Kutzneria* species are available in public databases, NCBI. The 3 complete genome sequences (1 contig) are merely from *Kutzneria albida* DSM43870^T^, *Kutzneria chonburiensis* SMC256^T^ and *Kutzneria* sp. CA-103260. The remaining are incomplete genome sequences (draft genome) of *Kutzneria buriramensis* DSM45791^T^ (65 contigs), *Kutzneria viridogrisea* NBRC15561^T^ (11 contigs), *Kutzneria kofuensis* DSM43851^T^ (5 contigs) and *Kutzneria* sp. 744 (534 contigs). The completeness assessment of the assemblies with BUSCO shows genome assemblies of very good quality excepted for *Kutzneria* sp. 744. In fact, they all have a BUSCO score higher than 98% without any missing genes and between 0 and 4 fragmented genes excepted for *Kutzneria* sp. 744 that has a BUSCO score of 87.3% with many missing genes and many fragmented genes. Furthermore, the BUSCO results at the annotation level show little difference with what was observed at the assembly level (> 98% for all annotation excepted for *Kutzneria* sp. 744 with 86.2% (Table 2). For genome comparison, all available genome sequences were retrieved and used for genome sequence comparison with that of *K. chonburiensis* through a circular map created by BLAST method (Fig. 2). This circular map shows gaps and low sequence similarity regions (pale color shades) indicating wide variable regions among the compared genomes. The results of this annotation exhibit the genome lengths between 9.87 and 11.96 Mbp, GC% of 69.86–70.6%, and CDS of 8894–11,066 genes. There are repeat regions within the genome sequences of *Kutzneria* sp. 744, *Kutzneria buriramensis*, *Kutzneria albida* and *Kutzneria viridogrisea* but not in *K. chonburiensis*, *K. kofuensis* and *Kutzneria* sp. CA-103260. To determine genome similarity among the *Kutzneria* species, the pairwise genome average nucleotide identity (ANI) values calculated by *JSpecies* were used as an index for genome comparisons (Table 1). This ANI comparison between the genome of *K. chonburiensis* with those of other species showed the results of < 95%, implying that *K. chonburiensis* does not belong to the same species with *Kutzneria* sp. 744 (89% ANI), *K. kofuensis* (88.6% ANI), *Kutzneria* sp. CA-103260 (87.6% ANI),* K. buriramensis* (87.42% ANI), *K. albida* (84.65% ANI) and *K. viridogrisea* (84.65% ANI). Based on phylogenomic analysis using the whole-genome multi-locus sequence typing (MLST), *Kutzneria* species formed a unique group in the family *Pseudonocardiaceae* in which *K. chonburiensis* is closely related to *Kutzneria* sp. 744, *K. kofuensis*, *Kutzneria* sp. CA-103260 and *K. buriramensis,* respectively and rather far from *K. viridogrisea* and *K. albida* (Fig. 3). In addition, functional roles of genes present in these genomes were annotated, assigned and categorized using RAST and tools in PATRIC (Pathosystems Resource Integration Center) server platform as seen in Table 2. It is noted that among the total ~ 10,000 annotated genes, nearly 60% of the genes encoding proteins with known functions have been assigned. The remaining (40%) have been predicted to code for hypothetical proteins. According to the RAST database, the majority of genes have been functionally classified into metabolism, protein processing, stress response, energy, DNA processing, RNA processing, cellular processing, membrane transport, cell envelops, regulation and cell signaling, and miscellaneous. By this analysis, the most abundant genes were distributed in the category of metabolism (Table 2). Further studying the clusters of orthologous groups of proteins (COGs: a family of orthologous protein-coding genes) within genomes of the *Kutzneria* was represented in a clustering heat map (Fig. 4). The orthologous protein-coding genes were assigned and mapped into 23 functional COG classes with their gene abundance (represented by colors) over the members of this group. It is noteworthy that *K. albida* and *K. viridogrisea* clearly displayed the most similar heat map patterns of COGs in the same clusters. These gene clusters belonged to (L) lipid transport and metabolism, (Q) secondary metabolites biosynthesis, transport and catabolism, (E) amino acid transport and metabolism, (F) nucleotide transport and metabolism, (H) coenzyme transport and metabolism, (J) translation, ribosomal structure and biogenesis, (B) chormatin structure and dynamics, (O) post-translational modification, protein turnover, chaperones, (L) replication, recombination and repair, (V) defense mechanisms, and (P) inorganic ion transport and metabolism. On the other hand, although *K. chonburiensis* was closest to *K. kofuensis*, *Kutzneria* sp. CA-103260, *K. buriramensis* and *Kutzneria* sp. 744, it showed the distinct pattern of COGs among them in which the genes were located mostly in the categories of (U) intracellular trafficking, secretion and vesicular transport, and (G) carbohydrate transport and metabolism, and some were identified to be (S) unknown function (Fig. 4).

**Table 1:** Genome assembly statistics and features annotated using the RAST tool kit system.

Strain	Genome size (Mbp)	GC (%)	Contig	Genome completeness (%)	Genome fragmented BUSCOs	Genome missing BUSCOs	CDS	rRNA	tRNA	Repeat region	ANI
*Kutzneria chonburiensis*	10.41	69.86	1	98.6	4	0	9564	9	74	0	100.00
*Kutzneria* sp. 744	11.64	69.81	534	87.3	26	19	10,978	5	80	3	89.00
*Kutzneria kofuensis*	10.44	70.49	5	99.7	1	0	9237	9	72	0	88.60
*Kutzneria* sp. CA-103260	11.60	70.12	1	100.0	0	0	10,295	12	67	0	87.60
*Kutzneria buriramensis*	11.96	70.35	65	99.5	2	0	10,923	6	78	6	87.42
*Kutzneria albida*	9.87	70.60	1	99.8	1	0	8828	9	57	1	84.65
*Kutzneria viridogrisea*	10.25	70.60	11	99.8	1	0	9233	9	57	3	84.65

**Table 2:** Functional annotations according to the subsystem analysis of the *Kutzneria* genomes show their genes distribution in different functional categories.

	*Kutzneria chonburiensis*	*Kutzneria* sp. 744	*Kutzneria kofuensis*	*Kutzneria* sp. CA-103260	*Kutzneria buriramensis*	*Kutzneria albida*	*Kutzneria viridogrisea*
Proteins with functional assignments	5454	6003	5513	5964	5879	5218	5291
Hypothetical proteins	4249	5369	3977	4593	5534	3883	4272
Proteins with PATRIC subsystem assignment	2241	2196	2260	2425	2423	2339	2388
Annotation completeness (%)	98.3	86.2	99.8	99.7	99.6	99.5	99.5
Annotation fragmented BUSCOs	4	22	1	0	2	1	1
Annotation missing BUSCOs	2	27	0	1	0	1	1
Subsystems							
Metabolism	1063	1011	1088	1220	1188	1142	1173
Protein processing	266	243	267	269	268	276	273
Stress response	190	181	191	194	194	204	208
Energy	345	329	335	356	366	315	327
DNA processing	92	113	101	95	110	75	75
Cellular processing	102	112	93	92	96	113	113
RNA processing	44	43	45	43	44	43	43
Membrane transport	46	75	49	51	57	67	69
Cell envelops	28	23	28	34	29	33	35
Regulation and cell signaling	41	44	41	46	46	41	42
Miscellaneous	24	22	22	25	25	30	30

Numbers in columns indicate the gene abundance.

### Secondary metabolite biosynthetic gene clusters (BGCs) of *Kutzneria chonburiensis* and the other species of the genus *Kutzneria*

**Figure 5:**
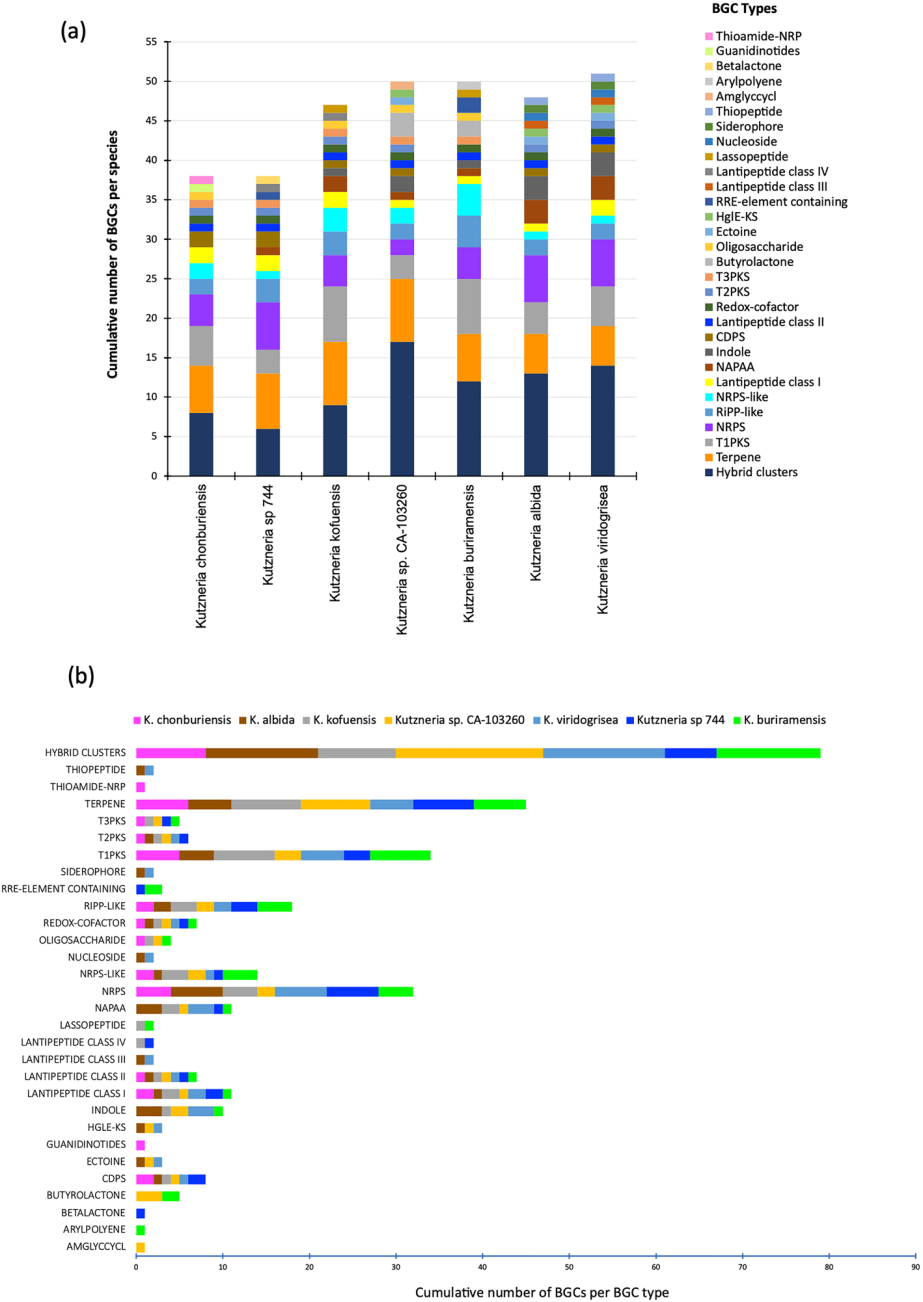
The 30 different types of secondary metabolites biosynthetic gene clusters (BGCs) in genomes of *Kutzneria* species. The proportion of different BGC types (**a**) and of same BGC type (**b**) among *Kutzneria chonburiensis*, *K. kofuensis*, *K. buriramensis*, *K. albida*, *K. viridogrisea*, *Kutzneria* sp.744 and *Kutzneria* sp. CA-103260.

**Figure 6:**
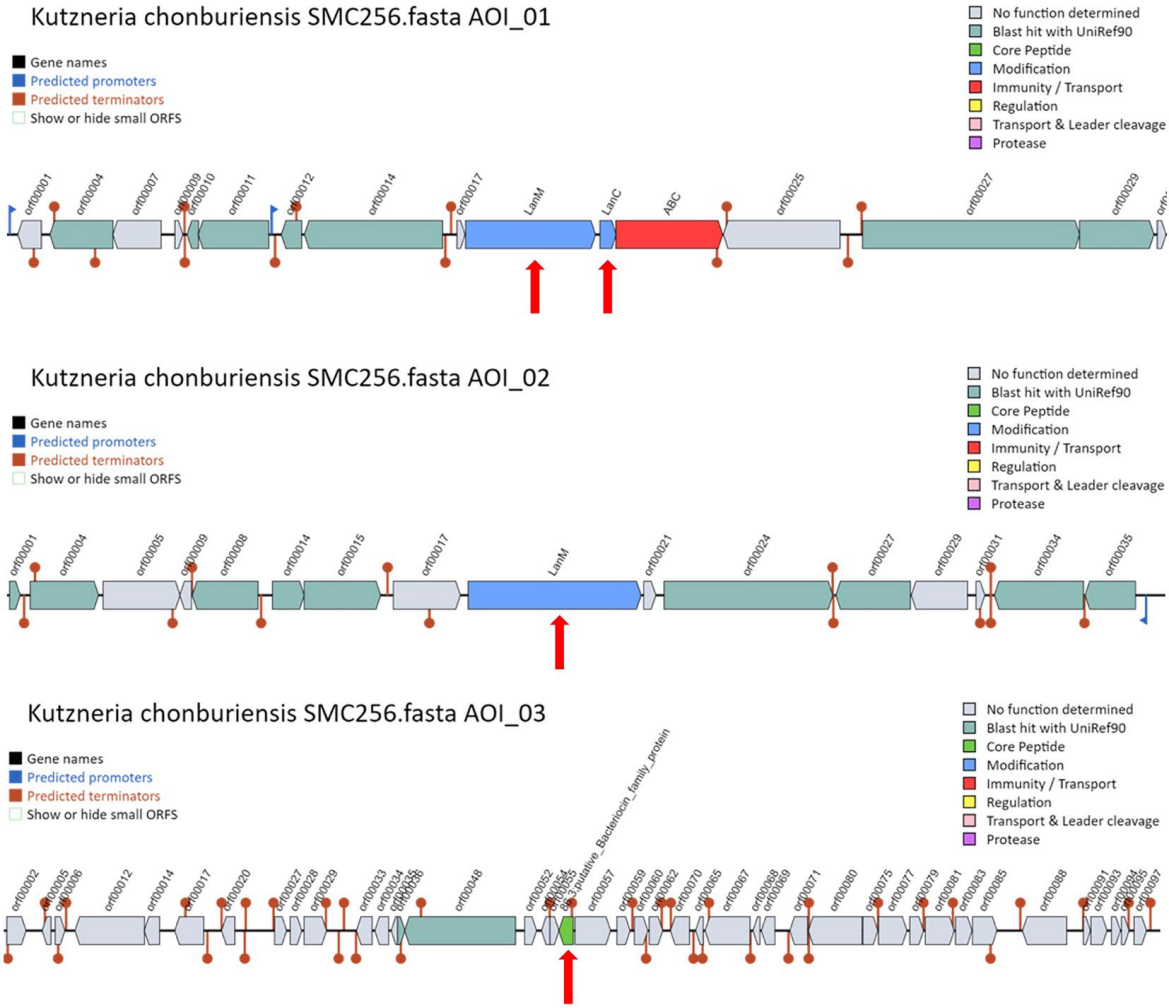
The 3 putative bacteriocin gene clusters of *Kutzneria chonburiensis* strain SMC256^T^ predicted by BAGEL 4.0 (AOI is the area of interest where the bacteriocin gene clusters had been identified by the program. AOI 1 and AOI 2 were best hit to lanthipeptide class II BGCs (LanM, LanC as indicated by red arrows) and AOI 3 was hit to a putative bacteriocin-family protein, as pointed by red arrow).

**Figure 7:**
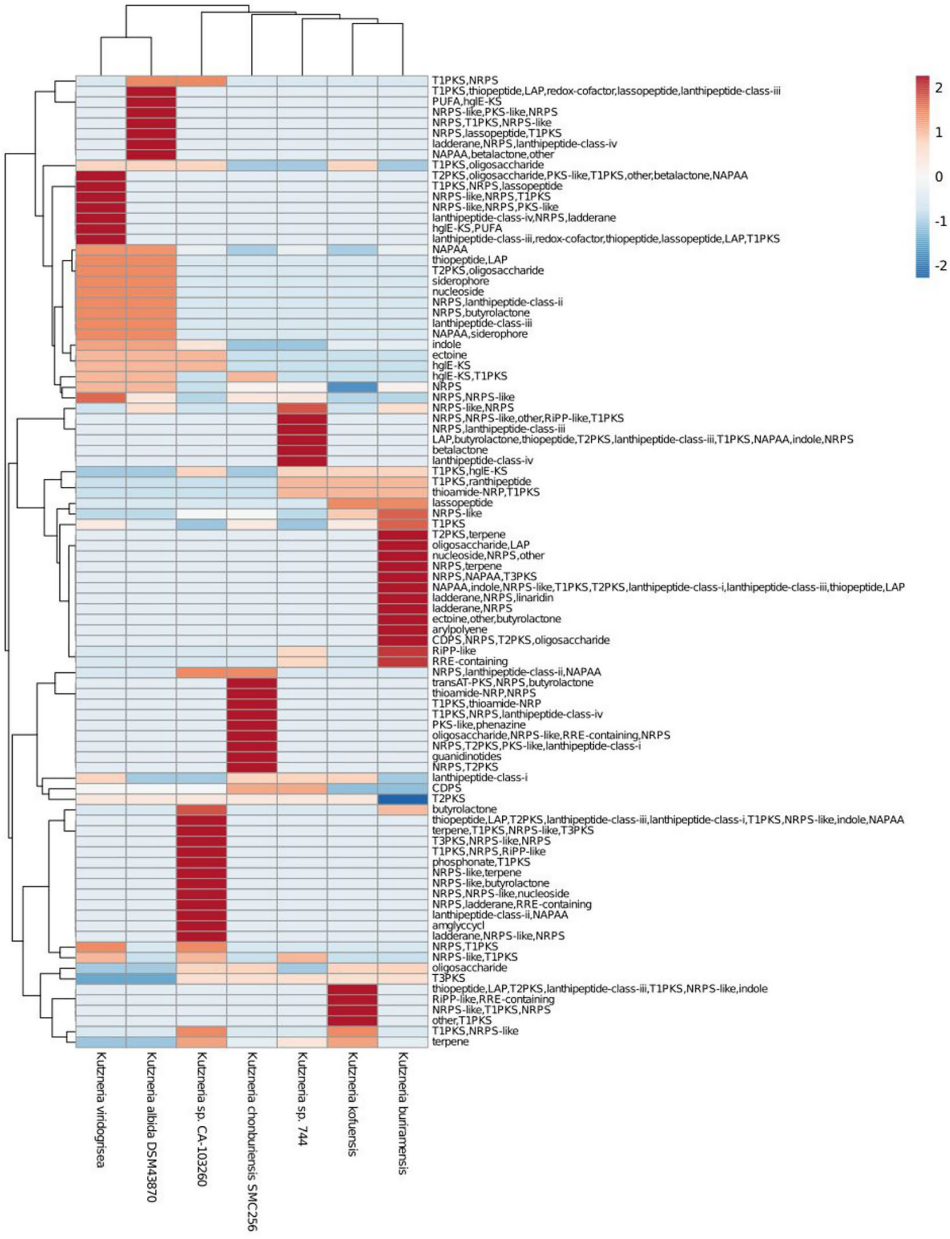
Heat map showing the abundance of BGCs responsible for production of putative secondary metabolites in *Kutzneria* genomes as predicted by antiSMASH. The color box represents numbers of individual cluster.

**Figure 8:**
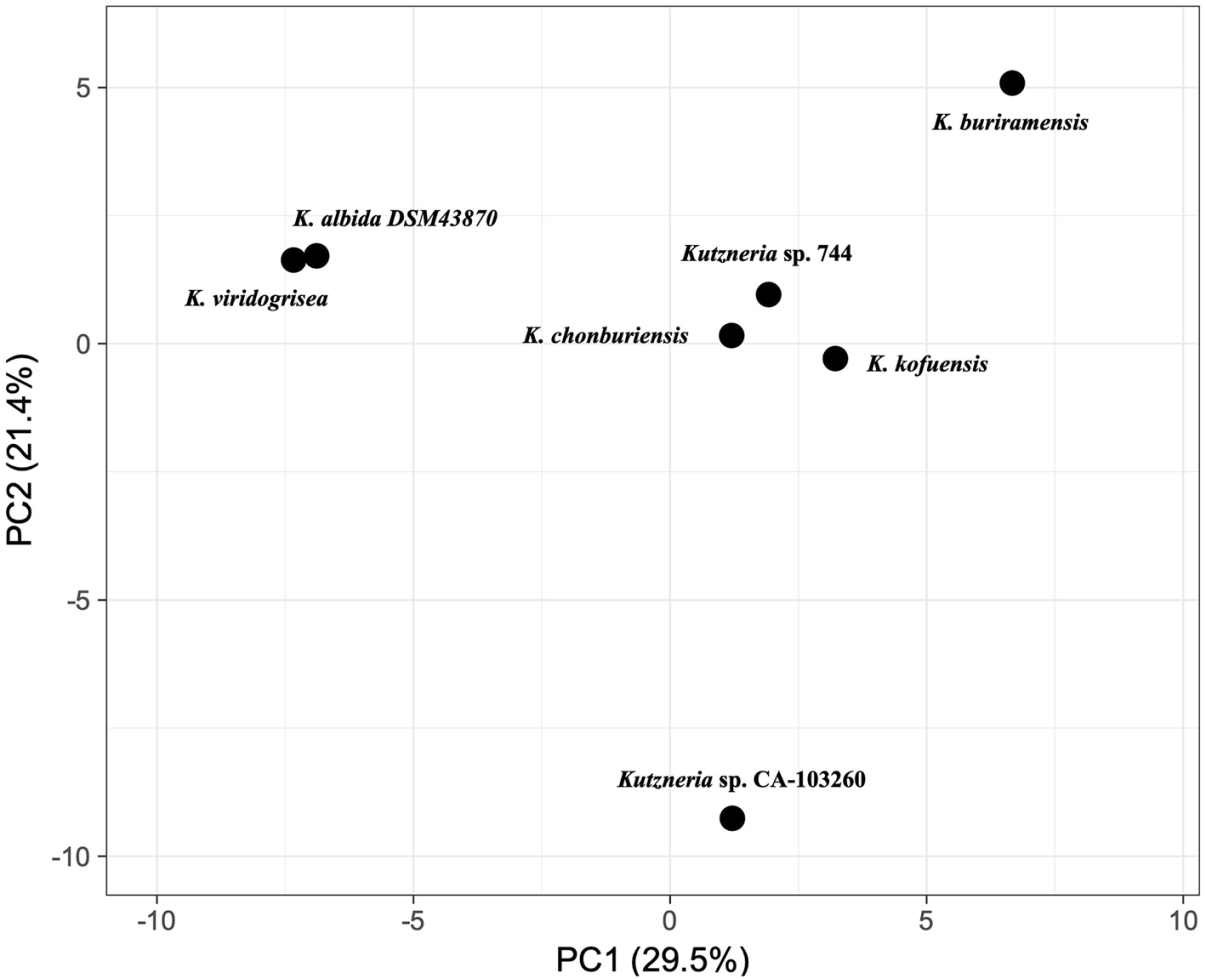
Principal component analysis (PCA) among *Kutzneria* species derived on the BGCs recovered through antiSMASH for their relationship.

**Figure 9:**
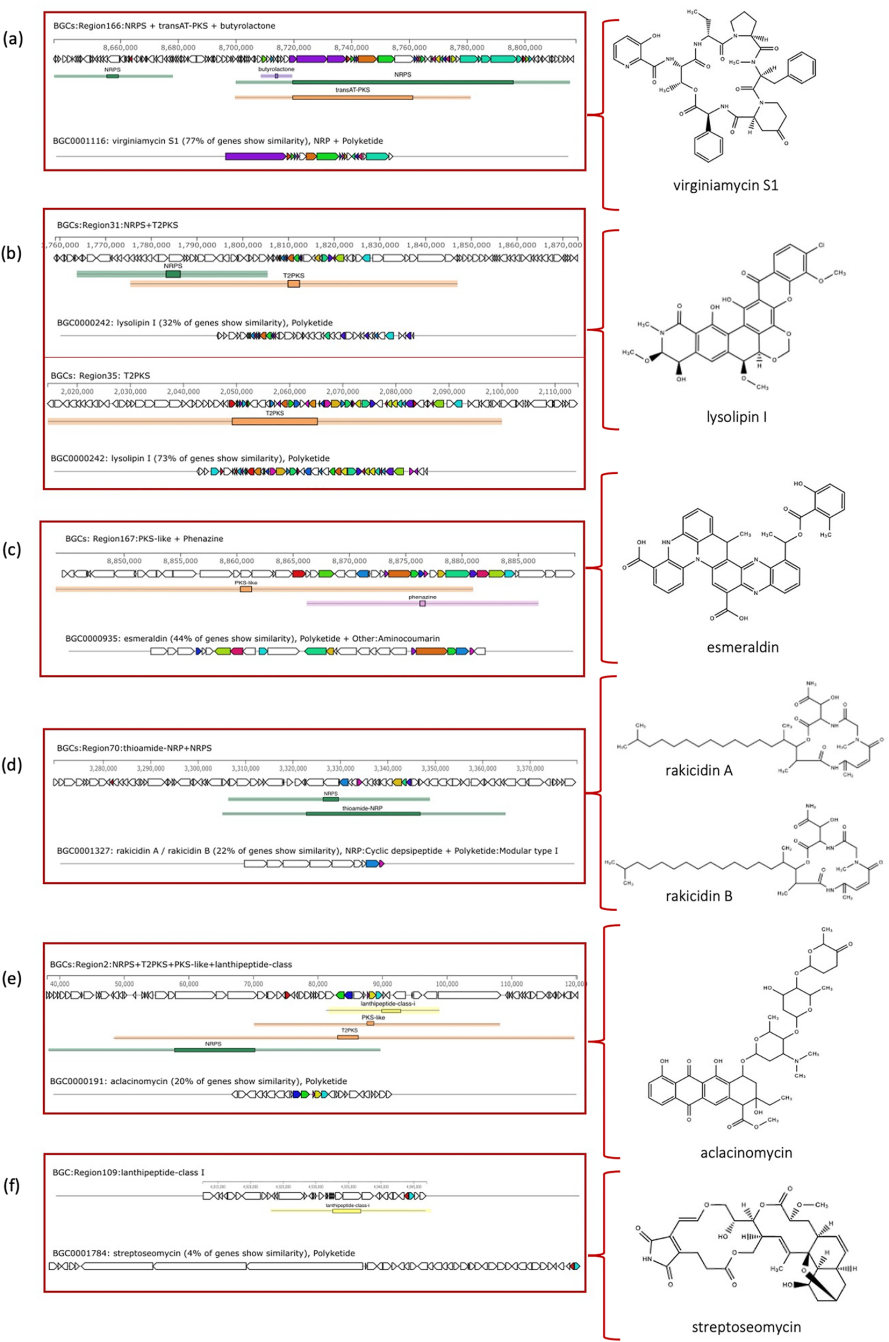
Putative species-specific antimicrobial/antitumor biosynthetic gene clusters in *Kutzneria chonburiensis* obtained by antiSMASH. The identified gene clusters were compared with known clusters in the MIBiG database to identify the putative compounds produced by these clusters. Gene clusters for (**a**) virginiamycin S1, (**b**) lysolipin I, (**c**) esmeraldin, (**d**) rakicidins A/B, (**e**) aclacinomycin, and (**f**) streptoseomycin.

To elucidate high potential genes involved in the secondary metabolite biosynthesis in bacterial genomes, antiSMASH version 6.0 has been used to rapid genome-wide identification, annotation and analysis of the secondary metabolite biosynthesis gene clusters (BGCs) in the *Kutzneria* spceis^15^. The results revealed that 322 gene clusters were identified and distributed among the *Kutzneria* genomes and grouped into 30 BGC types as seen in Fig. 5 and Supplementary Table S2. Among them, the hybrid clusters, terpene, Type I PKS (T1PKS), non-ribosomal peptide synthetase cluster (NRPS), other unspecified ribosomally synthesised and post-translationally modified peptide product cluster (RiPP-like), NRPS-like fragment (NRPS-like), Class I lanthipeptide clusters like nisin (Lantipeptide I), Class II lanthipeptide clusters like mutacin II (Lantipeptide II) and Redox-cofactors such as PQQ (Redox-cofactor) were widespread in all *Kutzneria* species, especially hybrid clusters, terpene, T1PKS and NRPS were the predominant BGCs of the *Kutzneria* genus (Fig. 5b). In fact, the largest number of BGCs in all analyzed species was the hybrid clusters (~ 25% in dark blue) and the second most abundant BGCs was the terpene compounds (~ 14% in orange) (Fig. 5a). Furthermore, out of 30 BGC types, 15 types were identified in the genome of *K. chonburiensis*, which include tRNA-dependent cyclodipeptide synthases (CDPS), pheganomycin-style protein ligase-containing cluster (guanidinotides), lantipeptide class I, lantipeptide class II, NRPS, NRPS-like, oligosaccharide cluster (oligosaccharide), redox-cofactor, RiPP-like, T1PKS, type II PKS (T2PKS), type III PKS (T3PKS), terpene, thioamide-containing non-ribosomal peptide (thioamide-NRP) and hybrid clusters (Fig. 5a). A small number of putative bacteriocins, mainly belonging to the class II lanthipeptide were also determined in the genome of *K. chonburiensis*, as shown in Fig. 6 and Supplementary Table S3. The heat map in Fig. 7 representing abundance and clustering of BGCs among the *Kutzneria* genomes revealed strain/species-specific biosynthetic gene clusters of each *Kutzneria* species (red boxes) and slightly separated members of the *Kutzneria* into 2 subgroups: *K. albida* and *K. viridogrisea* in the same group whereas *K. buriramensis*, *K. kofuensis*, *Kutzneria* sp. 744, *Kutzneria* sp. CA-103260 and *K. chonburiensis* in another group. Moreover, the principal component analysis (PCA) derived on BGCs data as shown in Fig. 8 also clearly supported that these *Kutzneria* species formed separate clusters (cluster 1: *K. viridogrisea* and *K. albida*; cluster 2: *K.chonburiensis*, *Kutzneria* sp. 744, *K. kofuensis*, *K. buriramensis* and *Kutzneria* sp. CA-103260) similarly to patterns of cladogram (Fig. 3) and clustering heat map (Fig. 7). Among all BGCs detected, 235 gene clusters were predicted directly through the antiSMASH to be responsible for biosynthesis of known metabolites (compounds). Geosmin, lankacidin C and hopene are common secondary metabolites found in all analyzed *Kutzneria* (see Supplementary Table S4). Interestingly, the *Kutzneria chonburiensis* genome possesses several specific putative BGCs among the genus that matched the known biosynthetic gene clusters in MIBiG at various % sequence similarity. These matched known BGCs are reported to be responsible for biosynthesis of virginiamycin S1 (77% similarity), lysolipin I (32% and 73% similarities), esmeraldin (44% similarity), rakicidins A and B (22% similarity), aclacinomycin (20% similarity) and streptoseomycin (4% similarity) (Fig. 9). Additionally, according to protein-blasting of these gene clusters against the NCBI database, we found the similar gene clusters located in other microorganisms, including *Micromonospora* sp. NBS 11–29 (40% similarity to gene cluster coding for putative virginiamycin), *Streptomyces auratus* AGR0001(58% similarity to genes coding for putative lysolipin), *Burkholderia ubonensis* MSMB2035(48% similarity to genes coding for putative esmeraldin), *Streptomyces guanduensis* CGMCC4.2022 (19% similarity to genes coding for putative lanthipeptide class II), *Streptomyces* sp. BK387 (25% similarity to genes coding for putative lanthipeptide class I) and *Saccharothrix tamanrassetensis* (21% similarity to genes coding for unidentified compound), respectively.

## Discussion

During the period of exploration of actinomycetes biodiversity and searching for new antimicrobials in Thailand, *Kutzneria chonburiensis* strain SMC256^T^ was isolated and taxonomically identified from mountain soil. Because it is the latest type-strain and the potential producer of novel secondary metabolites in the rare group of *Kutzneria*, the insights into this genome will be noteworthy to uncover the diversity and uniqueness of bioactive metabolites synthesis in this strain. In this study, we generated short- and long-reads using the Illumina sequencing and the Oxford Nanopore technology, respectively, and employed this hybrid sequencing technique to complete genome assembly of the strain SMC256^T^. In general, using only short-read sequencing strategy for genome assembly usually leads to an incomplete assembly of important segments of the genome. Supplementation of long reads can overcome these spontaneous errors and complete crucial details of the genome^17^. Hence, hybrid technique was applied to fill ambiguous gaps that exist in a draft genome sequence previously obtained from the short-read sequencing, as described especially in many actinomycetes genomes which carry complex repeated DNA segments^18^. In our case, the hybrid genome assembly improved contiguity and identified more expected genes. Indeed, using hybrid assembly, we were able to obtain circular completed genome (1 contig) while using Illumina short reads alone produced gaps in the assembly, resulted in discontinuous genome contigs (42 contigs) and made some missing annotated genes (CDS, RNA) as seen in supplementary Table S5. By the hybrid approach, the completed genome sequence of the strain SMC256^T^ revealed high G+C content (69.86%) and large genome size (10.41 Mbp) which is a typical characteristic of actinobacterial genomes^19^. Genome annotation also revealed that half of gene abundances are functionally assigned to the metabolism (1063 genes) probably encoding complex secondary metabolites. Moreover, we observed high numbers of hypothetical proteins (4249 genes), indicating that unknown proteins have not been identified yet (Table 2). Phylogenomic tree analysis of these *Kutzneria* based on MLST revealed the similar cladogram pattern to those obtained by clustering heatmaps of COGs and secondary metabolite BGCs analyses (antiSMASH). These results, in agreement with the PCA, obviously conveyed the consistency in close relationships among the *Kutzneria* species. *Kutzneria chonburiensis*, *Kutzneria* sp.744, *K. kofuensis*, *Kutzneria* sp. CA 103260 and *K. buriramensis* were grouped in a cluster whereas *Kutzneria viridogrisea* and *Kutzneria albida* were in another minor group in the same node of the genus *Kutzneria* (Figs. 3, 4, 7). Analysis of secondary metabolite gene clusters using bioinformatics tools showed that the most abundant BGCs found among genomes of the *Kutzneria* were “hybrid clusters” consisting of different types of BGCs which imply that they have a high potential for producing secondary metabolites with high structural diversity (Fig. 5). However, there were a number of clusters identified by antiSMASH with no similarity to the known BGCs in the databases (e.g. MIBiG), which were suggested to be orphan gene clusters. By identifying these genes, it is highly possible to discover novel secondary metabolites^20,21^. *Kutzneria chonburiensis* strain SMC256^T^ could be bioinformatically predicted to possess the unique gene clusters which did not exist in genomes of other *Kutzneria* (Fig. 7). The unique gene clusters include guanidinotides, hybrid clusters of (NRPS/T2PKS/PKS-like/Lanthipeptide class-I), (oligosaccharide/NRPS-like/RRE-containing/NRPS), (thioamide/NRP/NRPS), (transAT-PKS/NRPS/butyrolactone), (PKS-like/phenazine), (T1PKS/ thioamide-NRP), (NRPS/T2PKS) and (T1PKS/NRPS/lanthipeptide class-IV). Of the 9 unique gene clusters, 6 clusters were responsible for biosynthesis of known important biologically active secondary metabolites, including virginiamycin S1, lysolipin I, esmeraldin, rakicidin, aclacinomycin and streptoseomycin (Fig. 9). Virginiamycin S1, originally produced by *Streptomyces virginiae* in 1950s, is a cyclic depsipeptides antibiotic belonging to the Streptogramin family group B and shows a strong synergistic bactericidal activity (100-fold increase) against a wide range of Gram-positive bacteria, including multidrug-resistant bacteria when combination with virginamycin M1 (Streptogramin family group A). The extraordinary features of the virginiamycin biosynthesis is a simultaneous production of both M1 and S1 at a suitable ratio providing their maximum synergistic activity in the suppression of the protein biosynthesis in susceptible microorganisms^22,23^. Even though the detailed pathway of the Virginiamycin biosynthesis has not been clarified, at least four and twenty plausible genes were identified for Virginiamycin S and Virginiamycin M biosynthesis, respectively. Because virginiamycin is a close structural relative to pristinamycin (a member of the same Streptogramin family) they were proposed to share a common biosynthetic gene cluster^24^. The identified BGCs (NRPS/transAT-PKS/butyrolactone) of region 166 of strain SMC256^T^ by antiSMASH shared 77% sequence similarity of gene product to the best hit known functional BGCs of virginiamycin S1(NRPS/PKS) in MIBiG database (Fig. 9a). Lysolipin has strong antibacterial activity against a wide variety of multidrug-resistant pathogens and also has tumorstatic activity. This aromatic polyketide was first isolated in 1975 from *Streptomyces violaceoniger* Tü 96 and from *Streptomyces tendae* Tü 4042 in 1995^25,26^. Later, the complete lysolipin biosynthetic gene cluster was entirely sequenced and identified^27^. The cluster encodes a Type II polyketide synthases (T2PKSs), cyclases, methyltransferases, halogenase, amidotransferase, ferredoxin, transporter and regulatory proteins. Moreover, fifteen genes coding for enzymes involved in redox modifications of the polyketide precursor existed in the lysolipin biosynthetic gene cluster. Owing to this great number of oxidoreductases, lysolipin is among the most highly modified aromatic polyketides known so far^27^. The regions 31 and 35 of strain SMC256^T^, identified to contain NRPS/T2PKS by antiSMASH, showed 32% and 73% sequence similarities to the BGCs of lysolipin I (T2PKS) of *Streptomyces tendae* in MIBiG, respectively (Fig. 9b). Esmeraldins (A and B), dark green metabolites isolated from *Streptomyces antibioticus* Tü 2706 in 1988, are antimicrobial and antitumor metabolites^28^. A chemical core structure of esmeraldins consists of diphenazines, which show a wide range of bioactivities. The 24 putative genes in biosynthetic gene cluster of esmeraldin production were identified and involved in the complicated synthetic pathways of phenazine-1-carboxylic acid (PCA) and phenazine-1,6-dicarboxylic acid (PDA). Phenazines are electron shuttles that reduce molecular oxygen and generate toxic reactive oxygen species making them broadly inhibit growth of bacteria, fungi and parasites^29^. The BGCs (PKS-like/phenazine) of region 167 of the strain SMC256^T^ identified by antiSMASH showed 44% sequence similarity of gene to known functional BGCs of esmeraldin (PKS/aminocoumarin) in MIBiG (Fig. 9c). Rakicidins (A and B), a cyclic depsipeptide compound originally discovered from *Micromonospora* sp. and *Streptomyces* sp., display antitumor activity selectively to hypoxic cancer cells and stem-like leukemia cells^30,31^. The rakicidin B differs from rakicidin A by one additional methylene group in the lipid side chain and its cytotoxic activity (IC50 = 200 ng/ml) was less than that of rakicidin A (IC50 = 40 ng/ml). The biosynthetic pathways of rakicidins A and B, involved a hybrid PKS-NRPS biosynthetic gene clusters and being rather different from other types of rakicidins (e.g. rakicidin D), were elucidated in *Micromonospora* sp. M42 and *M. purpureochromogenes* NRRL B-2672^32^. The BGCs (thioamide-NRP/NRPS) of region 70 of strain SMC256^T^ identified by antiSMASH shared 22% sequence similarity of gene to known functional gene cluster of rakicidins A/B (NRP: cyclic depsipeptide/T1PKS) in MIBiG (Fig. 9d). Another anticancer/antibiotics drugs belonging to anthracycline are aclacinomycins, which were isolated from *Streptomyces galilaeus*^33^. The aromatic polyketide anthracyclines are able to penetrate and accumulate in high concentration in cancer cells in which they act on suppression of the DNA synthesis. It is used in treatments of acute myelogenous and lymphoblastic leukemia, malignant lymphoma, and gastric, lung, breast and ovarian cancers^34^. The biosynthesis of aclacinomycins, likely to anthracyclines biosynthetic pathways with some modification, was directed by a hybrid type II PKS/nonribosomal peptide synthetase (NRPS) system^35^. This complex biosynthesis comprised BGCs of Type II PKS, post-PKS tailoring enzymes and a multi-enzyme cascade including non-ribosomal peptide (NRP) assembly steps. The region 2 of strain SMC256^T^, identified to contain NRPS/T2PKS/PKS-like/lanthipeptide class-I by antiSMASH, showed 20% sequence similarities to known functional BGCs of aclacinomycin (PKS) of *Streptomyces galilaeus* in MIBiG (Fig. 9e). The last secondary metabolite predicted in *Kutzneria chonburiensis* was streptoseomycin. Streptoseomycin, discovered in *Streptomyces seoulensis* in 2018, is a rare macrodilactone belonging to a small tricyclic macrolactone family with only four members discovered to date. This compound exhibited a potent inhibitory action against 8 microaerophilic bacteria and particularly most highly effective against *Helicobacter pylori*^36^. The biosynthesis of streptoseomycin was identified and managed by type I PKS gene cluster (76 kB) haboring giant genes that encode PKS megaenzymes^36^. The BGC (lanthipeptide class I) of region 109 of strain SMC256^T^ identified by antiSMASH shared 4% sequence similarity of gene to known functional BGCs of streptoseomycin (polyketide) in MIBiG database (Fig. 9f). The abovementioned technologies of hybrid sequencing-assembly, and data analytics of BGCs in genomes would be a crutial alternative approach to accelerate the discovery of new bioactive secondary metabolites hidden in the *Kutzneria*. To the best of our knowledge, only nine bioactive metabolites have been investigated in genus *Kutzneria*, of which four kutznerides (cyclic depsipeptides showing antifungal activity) were isolated from *Kutzneria* sp. 744^37^, aculeximycin (a macrolide being active against both bacteria and fungi) and huimycin (a pyrrolopyrimidine compound with a broad spectrum of biological activities) were from *Kutzneria albida*^16^, epemicins A and B (macrolides antibiotics against methicillin resistant *Staphylococcus aureus* (MRSA)) were extracted from *Kutzneria* sp. CA-10326^6^, and the last one, phenol, 2,4-bis (1,1-dimethylethyl) which exhibits antifungal activity were isolated from *Kutzneria* sp. strain TS II^38^. Moreover, *Kutzneria* is still one of an underexplored genus, which shows great individual variation in terms of the total number of BGCs, therefore, the need of using genome mining as an aid to guide structural elucidation of novel compounds would improve the frequencies of drug discovery in this rare actinomycete effortlessly.

In conclusion, genomic variation of *Kutzneria chonburiensis* strain SMC256^T^ in comparison with other strains/species in the genus has been demonstrated in accordance with the comparative genomics studies. Numerous BGCs of this strain have been shown to be both strain-specific and unidentified, which support the fact that microbes carry out specialized metabolic tasks exclusively for survival in particular ecological environment and potentially construct different metabolic routes for new bioactive secondary metabolite production. These findings contribute the future effective/productive effort to use integration of high throughput screening and bioinformatic based approaches to uncover, target and isolate new bioactive metabolites from the high rich sources of bioactive molecules including *Kutzneria* species and other rare actinobacteria.

## Methods

### Bacterial strain and cultivation condition

*Kutzneria chonburiensis* strain SMC256^T^ was isolated and taxonomically identified as described previously^39^. Briefly, it was isolated from a soil sample collected in a mountain forest in Chonburi province, Thailand. The sample was taken from the organic layer of the sandy soil with pH of 5.9, and kept at − 20 °C before being air-dried at 37 °C for 7 days. The strain SMC256^T^ was isolated on humic acid-salts vitamin agar supplemented with cycloheximide and nystatin. A pure culture was preserved by freezing at − 80 °C in glycerol (10% v/v) until use.

### Genomic DNA extraction

Genomic DNA (gDNA) of *Kutzneria chonburiensis* SMC256^T^ was extracted as stated by a modified method of Saito and Miura^40^ from cells grown in glucose-yeast extract broth at 28 °C, 250 rpm for 5 days. Freeze-dried cells were lysed using grinding with mortar and pestle, instead of lysozyme. The quantity and molecular weight of extracted genomic DNA were measured using the Denovix fluorometer (DeNovix Inc., DE, USA) and Agilent 2100 bioanalyzer (Agilent, CA, USA).

### Whole genome sequencing

The genomic DNA library of *Kutzneria chonburiensis* strain SMC256^T^ was prepared using the QIAGEN FX kit (Qiagen, USA). Briefly, gDNA was fragmented using enzymatic reaction and cleaned with magnetic beads. An adaptor index was ligated to the fragmented DNA. Quality and quantity of the indexed libraries were measured using Agilent 2100 Bioanalyzer and Denovix fluorometer and pooled in equimolar quantity. Cluster generation and paired-end 2 × 250 nucleotide read sequencing were performed on Illumina MiSeq sequencer. The sequencing process was carried out at the Omics Sciences and Bioinformatics Center, Chulalongkorn University, Bangkok, Thailand. The same DNA samples were prepared for long-read sequencing with the Oxford Nanopore Technologies (ONT) Ligation library preparation kit in accordance with the manufacturer’s protocol with the addition of continuous gentle mixing during the ligation incubation step. The libraries were sequenced with the ONT MinION sequencer using rev C R9.4 flow cells. The sequencer was controlled with the MinKNOW v.2.2.12, and sequencing runs were scheduled for 48–60 h, and allowed to run until fewer than ten pores remained functional. Raw base called data was generated using Guppy v.2.3.5, and the raw data was uploaded to the Galaxy web-based bioinformatics analysis tools platform (usegalaxy.eu). The pipeline of Hybrid de novo genome assembly—Nanopore draft Illumina polishing was carried out. First, adapters for Nanopore reads were removed using Porechop (v. 0.2.3)^41^, and Canu (v1.5) was used to assemble the filtered subreads^42^. Next, Pilon (v1.2) was used to polish the assembly sequence and improve genomic analyses with Illumina short reads^43^. In fact, when executing Pilon, three rounds of polishing were run iteratively on the assemblies, with Illumina short reads mapped to the polished assemblies obtained from the previous round using BWA-MEM^44^. In addition, genome assembly of short (Illumina) reads data alone was attempted in this study. Adaptors and poor-quality reads were removed using fastp (version 0.23.2) with detect_adapter_for_pe and -q 20 options^45^. Subsequentially, filtered reads were used as an input for SPAdes (version 3.15.4), genome assembly program with default parameters^46^.

### Genome annotation and comparisons

The complete genome sequences of *Kutzneria chonburiensis* strain SMC256^T^ and other close relatives were downloaded from the National Center for Biotechnology Information (NCBI). The Rapid Annotations using Subsystems Technology (RAST) was used for annotation and functional assignment (protein-encoding sequences (CDS), rRNA and tRNA genes and subsystem) to the genes in the genomes^47^. The annotated protein sequences were also assigned to clusters of orthologous groups (COGs) of proteins using WebMGA^48^, and the resulted COGs were compared and visualized in a heat map created by ClusVis^49^. The genome assembly statistics were evaluated using QUAST^50^. In addition, completeness estimation of the genome annotations and assemblies was assessed through identification of Benchmarking Universal Single-Copy Orthologs (BUSCO v.5.1.2) using the actinobacteria class dataset odb10 which contains 356 BUSCO markers^51^. Pairwise genome average nucleotide identity (ANI) was calculated using a web server, JSpeciesWS^52^. Phylogeny for genome comparison was constructed according to multi-locus sequence analysis (MLSA) technique using autoMLST^53^.

### Identification of the putative secondary metabolite biosynthetic gene clusters

Putative secondary metabolite gene clusters (BGCs) in the genomes of *Kutzneria* were predicted using antiSMASH (v 6.0) with default settings^15^. The abundance of BGCs of *Kutzneria chonburiensis* was compared along with those of other species in this genus. The results were displayed in a heatmap generated as previously mentioned. Additionally, putative bacteriocins were identified in all analyzed genomes using the online web-server of BAGEL 4^10^.

## Supplementary Information

Supplementary Tables.

## Data Availability

All data are available under BioProject PRJNA835675, and the GenBank accession number for the genome is CP097263. Other data associated with the research are available as Supplementary Information file.
