# Supplementary material for: Comparative genome features and secondary metabolite biosynthetic potential of Kutzneria chonburiensis and other species of the genus Kutzneria
Source: Sci Rep. 2023 May 31;13:8794. doi: 10.1038/s41598-023-36039-x (PMC10232511; doi:10.1038/s41598-023-36039-x)
Supplement: Supplementary file 1 — Supplementary Tables. [file 41598_2023_36039_MOESM1_ESM.pdf]

Comparative genome features and secondary metabolite biosynthetic potential of *Kutzneria chonburiensis* and other species of the genus *Kutzneria*

Manee Chanama[1*], Pinidphon Prombutara[2] and Suchart Chanama[3]

[1] Department of Microbiology, Faculty of Public Health, Mahidol University, Bangkok 10400, Thailand.

[2] Omics Sciences and Bioinformatics Center, Faculty of Science, Chulalongkorn University, Bangkok, 10330, Thailand.

[3] Department of Biochemistry, Faculty of Science, Chulalongkorn University, Bangkok 10330, Thailand.

* Author for correspondence: Manee Chanama, E-mail address: manee.cha@mahidol.ac.th

Tel.: +66 2354 8528; Fax: +66 2354 8538

Table S1. Strains of *Kutzneria* species that have been publicly available (August 24, 2022).

No.	Species	Strain	Type Strain	Genome sequence			Public Database		Original Country
				Complete	Incomplete (contigs)	Genome size (Mbp)	Database	Year	
1	*Kutzneria albida*	DSM43870	✓	✓		9.88	WGS-NCBI	1968	Japan
2	*Kutzneria buriramensis*	A-T1846, DSM45791	✓		✓ (65)	11.97	WGS-NCBI	2013	Thailand
3	*Kutzneria chonburiensis*	SMC256	✓	✓		10.41	WGS-NCBI	2015	Thailand
4	*Kutzneria viridogrisea*	DSM43850	✓		✓ (11)	10.25	WGS-NCBI	1966	Japan
5	*Kutzneria kofuensis*	DSM43851	✓		✓ (5)	10.44	WGS-NCBI	1969	Japan
6	*Kutzneria* sp.	CA-103260		✓		11.61	WGS-NCBI	1998	Panama
7	*Kutzneria* sp.	744			✓ (534)	11.65	WGS-NCBI	2014	Norway
8	*Kutzneria* sp.	A14					16S rRNA	2019	India
9	*Kutzneria* sp.	TSII					16S rRNA	2019	India
10	*Kutzneria* sp.	TM-S116					16S rRNA	2018	Korea
11	*Kutzneria* sp.	TM-B149					16S rRNA	2018	Korea
12	*Kutzneria* sp.	RMD-3Y-3-1					16S rRNA	2014	Korea
13	*Kutzneria* sp.	RDB-177					16S rRNA	2014	Japan
14	*Kutzneria* sp.	306G04					16S rRNA	2003	Japan
15	*Kutzneria* sp.	L1988					16S rRNA	2012	U.S.A

Table S2. Biosynthetic gene clusters (322 BGCs) identified in the genomes of *Kutzneria* species by antiSMASH version 6.0 (number of gene clusters is present in the table).

Kutzneria chonburiensis (KC), *Kutzneria* sp. 744 (K744), *K. kofuensis* (KK), *Kutzneria* sp. CA-103260 (KCA), *K. buriramensis* (KB), *K. viridogrisea* (KV), and *Kutzneria albida* (KA)

BGCs	Description	KC	K744	KK	KCA	KB	KA	KV
1. Amglyccycl	Aminoglycoside/ aminocyclitol cluster	0	0	0	1	0	0	0
2. Arylpolyene	Aryl polyene cluster	0	0	0	0	1	0	0
3. Betalactone	Beta-lactone containing protease inhibitor	0	1	0	0	0	0	0
4. Butyrolactone	Butyrolactone cluster	0	0	0	3	2	0	0
5. CDPS	tRNA-dependent cyclodipeptide synthases	2	2	1	1	0	1	1
6. Ectoine	Ectoine cluster	0	0	0	1	0	1	1
7. Guanidinotides	Pheganomycin-style protein ligase-containing cluster	1	0	0	0	0	0	0
8. HglE-KS	Heterocyst glycolipid synthase-like PKS	0	0	0	1	0	1	1
9. Indole	Indole cluster	0	0	1	2	1	3	3
10. Lantipeptide class I	Class I lanthipeptide clusters like nisin	2	2	2	1	1	1	2
11. Lantipeptide class II	Class II lanthipeptide clusters like mutacin II	1	1	1	1	1	1	1
12. Lantipeptide class III	Class III lanthipeptide clusters like labyrinthopeptin	0	0	0	0	0	1	1
13. Lantipeptide class IV	Class IV lanthipeptide clusters like venezuelin	0	1	1	0	0	0	0
14. Lassopeptide	Lasso peptide cluster	0	0	1	0	1	0	0
15. NAPAA	Non-alpha poly-amino acids like e-Polylysin	0	1	2	1	1	3	3
16. NRPS	Non-ribosomal peptide synthetase cluster	4	6	4	2	4	6	6
17. NRPS-like	NRPS-like fragment	2	1	3	2	4	1	1
18. Nucleoside	Nucleoside cluster	0	0	0	0	0	1	1
19. Oligosaccharide	Oligosaccharide cluster	1	0	1	1	1	0	0
20. Redox-cofactor	Redox-cofactors such as PQQ	1	1	1	1	1	1	1
21. RiPP-like	Other unspecified ribosomally synthesised and post-translationally modified peptide product (RiPP) cluster	2	3	3	2	4	2	2
22. RRE-containing	RRE-element containing cluster	0	1	0	0	2	0	0
23. Siderophore	Siderophore cluster	0	0	0	0	0	1	1
24. T1PKS	Type I PKS (Polyketide synthase)	5	3	7	3	7	4	5
25. T2PKS	Type II PKS	1	1	1	1	0	1	1
26. T3PKS	Type III PKS	1	1	1	1	1	0	0
27. Terpene	Terpene	6	7	8	8	6	5	5
28. Thioamide-NRP	Thioamide-containing non-ribosomal peptide	1	0	0	0	0	0	0
29. Thiopeptide	Thiopeptide cluster	0	0	0	0	0	1	1
30. Hybrid clusters	Others	8	6	9	17	12	13	14
Total		38	38	47	50	50	48	51

Table S3. Putative bacteriocin BGCs predicted in the genus *Kutzneria* by BAGEL 4.0.

Kutzneria chonburiensis (KC), *Kutzneria* sp. 744 (K744), *K. kofuensis* (KK), *Kutzneria* sp. CA-103260 (KCA), *K. buriramensis* (KB), *K. viridogrisea* (KV), and *Kutzneria albida* (KA)

KC	K744	KK	KCA	KB	KV	KA
Lanthipeptide class II	Lanthipeptide class II	Lanthipeptide class IV	GE2270	Sactipeptides	Lanthipeptide class I	Bottromycin
Lanthipeptide class II	LAPs	Bacteriocin family protein (putative)	Bacteriocin family protein (putative)	Thiomuracin A	Lanthipeptide class II	Lanthipeptide class II
Bacteriocin family protein (putative)	Sactipeptides	Lanthipeptide class II	Lanthipeptide class II	Sactipeptides	Lanthipeptide class IV	Thiomuracin
	Thiomuracins	Thiomuracin	Lanthipeptide class II	Bacteriocin family protein (putative)	Linocin M18	Lanthipeptide class IV
	Bacteriocin family protein (putative)	Chaxapeptin	LAPs	Sactipeptides	Lanthipeptide class II	Lanthipeptide class I
		Colicin	Sactipeptides	Sactipeptides	Thiomuracins	LAPs
			Sactipeptides	Colicin	Lanthipeptide class I	Bottromycin
			Cinnamycin	LAPs	Bottromycin	Bacteriocin family protein
				Lanthipeptide class II	Lanthipeptide class IV	Lanthipeptide class II
				Chaxapeptin		
				Linaridin		

Table S4. Summary of predicted known secondary metabolites derived from *Kutzneria* biosynthetic gene clusters.

Kutzneria chonburiensis (KC), *Kutzneria* sp 744 (K744), *K. kofuensis* (KK), *Kutzneria* sp. CA-103260 (KCA), *K. buriramensis* (KB), *K. viridogrisea* (KV), and *Kutzneria albida* (KA)

** metabolites produced in all analyzed *Kutzneria* species

* metabolites produced only in *Kutzneria chonburiensis*

KC	K744	KB	KA	KV	KCA	KK
aclacinomycin *	aldgamycin J<br>aldgamycin K<br>aldgamycin P<br>aldgamycin E	anantin C	2-methylisoborneol	2-methylisoborneol	acarbose	aculeximycin
alkyl-O-dihydrogeranyl-methoxyhydroquinones	alkyl-O-dihydrogeranyl-methoxyhydroquinones	arixanthomycin A<br>arixanthomycin B<br>arixanthomycin C	aculeximycin	aculeximycin	aculeximycin	alkyl-O-dihydrogeranyl-methoxyhydroquinones
ashimides	blasticidin S	atratumycin	aldgamycin J<br>aldgamycin K<br>aldgamycin P<br>aldgamycin E	allocyclinone	aculeximycin	anantin C
borrelidin	butyrolactol A	butyrolactol A	allocyclinone	amipurimycin	avoparcin	ansamitocin P-3
butyrolactol A	chlorizidine A	cadaside A<br>cadaside B	amychelin	amychelin	azalomycin F3a	arsono-polyketide
chlorizidine A	cyphomycin	clifednamide A	ashimides	ashimides	BE-54017	butyrolactol A
diisonitrile antibiotic SF2768	diisonitrile antibiotic SF2768	colabomycin E	AT2433-A1	AT2433-A1	bleomycin	chloramphenicol
elaiophylin	divergolide A<br>divergolide B<br>divergolide C<br>divergolide D	cyphomycin	atratumycin	atratumycin	chlorizidine A	chlorizidine A
esmeraldin *	elaiophylin	daptomycin	azicemicin B	aurantimycin A	chondrochloren A	collismycin A
friulimicin A<br>friulimicin B<br>friulimicin C<br>friulimicin D	enduracidin	elaiophylin	borrelidin	azicemicin B	chromomycin A3	divergolide A<br>divergolide B<br>divergolide C<br>divergolide D
geosmin **	geosmin **	FD-594	citrulassin A	borrelidin	cinnamycin	ECO-02301
glycinocin A	hopene **	fortimicin	cyphomycin	citrulassin A	colabomycin E	elaiophylin
herboxidiene	kutzneride 2	geosmin **	diisonitrile antibiotic SF2768	cyphomycin	cyphomycin	erdasporine A<br>erdasporine B<br>erdasporine C
hopene **	lankacidin C **	hopene **	ectoine	diisonitrile antibiotic SF2768	ectoine	fortimicin
JBIR-126	maduropeptin	JBIR-126	erdasporine A<br>erdasporine B<br>erdasporine C	ectoine	elaiophylin	frankiamicin
lankacidin C **	malacidin A<br>malacidin B	kosinostatin	Ery-9 / Ery-6 / Ery-8 / Ery-7 / Ery-5 / Ery-4 / Ery-3	erdasporine A<br>erdasporine B<br>erdasporine C	erdasporine A<br>erdasporine B<br>erdasporine C	geosmin **
lysolipin I *	mannopeptimycin	kutzneride 2	fortimicin	Ery-9 / Ery-6 / Ery-8 / Ery-7 / Ery-5 / Ery-4 / Ery-3	fluvirucin B2	guadinomine
maduropeptin	meridamycin	lankacidin C **	foxicins A-D	fortimicin	fortimicin	herboxidiene

mannopeptimycin	pyrrolomycin A<br>pyrrolomycin B<br>pyrrolomycin C<br>pyrrolomycin D	lavendiol	geosmin **	foxicins A-D	geosmin **	hopene **
meridamycin	quartromicin A1	limazepine C<br>limazepine D<br>limazepine E<br>limazepine F<br>limazepine A	hopene **	geosmin **	herboxidiene	lankacidin C **
phoslactomycin B	scabichelin	macrotermycins	JBIR-34 / JBIR-35	hopene **	hopene **	maklamicin
rakicidin A*<br>rakicidin B *	sceliphrolactam	marformycin A<br>marformycin B<br>marformycin C<br>marformycin D<br>marformycin E<br>marformycin F	JBIR-76 / JBIR-77	JBIR-34 / JBIR-35	hydroxysporine	meridamycin
scabichelin	SF2575	mediomycin A	K-252a	JBIR-76 / JBIR-77	ishigamide	miharamycin A<br>miharamycin B
SF2575	stenothricin	meoabyssomicin<br>abyssomicin	kutzneride 2	K-252a	kendomycin	naphthomycin A
streptoseomycin *	xantholipin	meridamycin	lankacidin C **	kutzneride 2	kirromycin	rifamycin
surugamide A<br>surugamide D		napyradiomycin	lipopeptide 8D1-1<br>lipopeptide 8D1-2	lankacidin C **	lankacidin C **	scabichelin
virginiamycin S1 *		scabichelin	macrotetrolide	lipopeptide 8D1-1<br>lipopeptide 8D1-2	leinamycin	sceliphrolactam
		SF2575	meilingmycin	macrotetrolide	meridamycin	SF2575
		spiramycin	nataxazole	meilingmycin	microsclerodermin	sporolide A<br>sporolide B
		stenothricin	oxytetracycline	nataxazole	nataxazole	stenothricin
		tiacumicin B	pyrrolomycin A<br>pyrrolomycin B<br>pyrrolomycin C<br>pyrrolomycin D	oxytetracycline	oxalomycin B	stenothricin
		toxoflavin fervenulin	pyxidicycline A<br>pyxidicycline B	pyrrolomycin A<br>pyrrolomycin B<br>pyrrolomycin C<br>pyrrolomycin D	phoslactomycin B	tomaymycin
		WS9326	rifamorpholine A<br>rifamorpholine B<br>rifamorpholine C<br>rifamorpholine D<br>rifamorpholine E	pyxidicycline A<br>pyxidicycline B	prejadomycin<br>rabelomycin<br>gaudimycin C<br>gaudimycin D UWM6<br>gaudimycin A	toxoflavin<br>fervenulin
		xiamycin	sceliphrolactam	rifamorpholine A<br>rifamorpholine B<br>rifamorpholine C<br>rifamorpholine D<br>rifamorpholine E	scabichelin	WS9326
			toyocamycin	sceliphrolactam	SF2575	xiamycin
			α-lipomycin	SF2575	telomycin	

				toyocamycin	thienodolin	
				α-lipomycin	toxoflavin<br>fervenulin	
					ulleungmycin	
					WS9326	

Table S5. Summary of draft and hybrid genome assemblies statistics and features annotated in *Kutzneria chonburiensis* strain SMC256^{T}.

Genome assembly method	Contig	CDS	rRNA	tRNA	Repeat region
Illumina short reads draft assembly	42	9,414	3	74	1
Hybrid assembly	1	9,564	9	74	0
